# Investigation of the cardioprotective potential of dantrolene in mitigating arsenic-induced cardiac dysfunction in rats: Cardioprotective potential of dantrolene in cardiac dysfunction

**DOI:** 10.3724/abbs.2025193

**Published:** 2025-10-15

**Authors:** Chuncui Chen, Ruoxi Chen, Xueting Guo, Lei Huang, Kuican Liu, Wenrong Shi, Caiyun Zhang, Kunxuan Liu, Huan Liu, Shanshan Dong, Guilin Lu, Wenjuan Qin

**Affiliations:** Department of Ultrasound Medicine the First Affiliated Hospital of Shihezi University Shihezi 832008 China

Arsenic, a toxic metalloid, exists in organic or inorganic states within the Earth’s seawater, river water, soil, atmosphere, food sources, and diverse biological tissues [1]. It poses a threat to the health of hundreds of millions of people globally [2]. Arsenic exposure has toxic effects on the cardiovascular system of organisms, thus endangering human health [3]. Research has indicated that the harmful effect of arsenic exposure on the heart is associated with abnormal calcium handling in myocardial cells [4]. The cardiac ryanodine receptor type 2 (RyR2) is a primary channel involved in the surface of the endoplasmic reticulum in cardiac myocytes that regulates the release of Ca ^2+^ during the systolic phase [5]. The integrity of its function is crucial for maintaining calcium homeostasis in cardiac myocytes. However, when myocardial tissue is damaged and undergoes pathological changes, the spatial structure of the RyR2 protein becomes unstable and becomes excessively activated, thereby triggering Ca ^2+^ leakage [6].

Dantrolene (Dan), which serves as a stabilizer of RyR1, is frequently employed in clinical settings for the treatment of malignant hyperpyrexia and relieves spastic muscle tension [7]. Previous studies have demonstrated that dantrolene also has a stabilizing effect on RyR2 [8]. Research has shown that dantrolene can prevent calcium leakage in myocardial cells by stabilizing the tertiary structure of the RyR2 protein and thereby inhibiting the pathological hyperactivity of RyR2 [9]. Therefore, this study hypothesizes that dantrolene, by virtue of this stabilizing effect, can alleviate myocardial injury caused by arsenic exposure to some extent and plays a role in protecting cardiac function. For this purpose, we established an arsenic exposure model and a Dan intervention arsenic exposure model to verify the protective effect of Dan on the myocardial tissue and cardiac function of arsenic-exposed rats.

Forty 12-week-old male Sprague-Dawley (SD) rats [SPF (Beijing) Biotechnology Company, Beijing, China; license: SCXK-2024-0001] of specific pathogen-free (SPF) grade were purchased and acclimated in isolation rooms at the Experimental Animal Center of Shihezi University for one week. After confirming no abnormalities, the rats were randomly assigned to 5 groups with 8 rats in each group: the control (Con) group, negative control (NC) group, arsenic exposure (As _2_O _3_) group, low-dose dantrolene (Dan-L) group and high-dose dantrolene (Dan-H) group. The rats in the As _2_O _3_, Dan-L and Dan-H groups received intraperitoneal injections of 5 mg/kg/day As _2_O _3_ (Beijing SL Pharmaceutical Company, Beijing, China) for 7 consecutive days. The rats in the NC, Dan-L and Dan-H groups subsequently received intraperitoneal injections of dantrolene sodium (Solarbio, Beijing, China) for 7 consecutive days at doses of 0, 5 and 10 mg/kg/day, respectively. Meanwhile, the rats in the Con group were administered i.p. injections of physiological saline. After the rat models in each group were established, indwelling needles were placed in the femoral veins of the rats to establish venous access. The myocardial contrast echocardiography (MCE) images of the rats were obtained via the SonoVue contrast agent (Bracco Suisse SA, Geneva, Switzerland) and the EPIQ7C ultrasound diagnostic instrument (Philips, Eindhoven, Netherlands). The images were subsequently processed offline with the aid of QLAB software. The time-intensity curve (TIC) was plotted, and key parameters such as peak intensity (PI), wash-in slope (WIS), the product of PI and WIS (WIS × PI), and the area under the curve (AUC) were automatically determined. Blood was drawn from the tail vein for sample collection to measure the levels of myocardial injury-related markers in the serum. The rats were subsequently sacrificed to harvest myocardial samples. Immunofluorescence staining was conducted using an anti-CD31 antibody (Boster, Wuhan, China) to detect the expression of CD31. Morphological changes in myocardial cells were observed via electron microscopy. The Animal Science College Bioethics Committee at Shihezi University granted approval for the experimental study (approval No. A2025-558).

MCE can be employed in flash scintigraphy imaging mode to utilize microbubbles for real-time observation of myocardial vascular perfusion, thus objectively evaluating the myocardial blood supply situation [10]. The experimental data indicated that the WIS and WIS × PI in the As _2_O _3_ group were lower than those in the Con group ( *P*  < 0.05). The above experimental results suggest that arsenic exposure could lead to a decrease in both myocardial blood volume and myocardial perfusion velocity in rats, which implies a decline in myocardial vascular perfusion function. This may be related to the fact that arsenic exposure can directly cause toxicity to capillaries [11]. In the rats treated with Dan, all the above-mentioned index parameters were greater than those in the As _2_O _3_ group. Moreover, with the difference in Dan dosage, the magnitude of improvement in these parameters also varied, showing a certain dose-related correlation ( *P*  < 0.05; Table 1). These findings indicate that different doses of Dan can improve the impaired myocardial microcirculation blood perfusion in rats caused by arsenic exposure to varying degrees.

**Table:** **Table 1** Comparison of MCE results among different groups ( *n* = 8)

Item	Con	NC	As _2_O _3_	Dan-L	Dan-H	*F*	*P*
PI (dB)	131.50 ± 5.13	132.81 ± 8.49	24.97 ± 3.65*	57.19 ± 4.25* ^,#^	71.14 ± 3.86* ^,#,†^	622.76	< 0.001
WIS (dB/s)	34.28 ± 3.71	32.79 ± 3.59	8.19 ± 1.68*	15.32 ± 2.12* ^,#^	21.95 ± 2.91* ^,#,†^	117.92	< 0.001
WIS × PI (dB ^2^/s)	4518.16 ± 621.04	4378.33 ± 726.19	202.12 ± 40.88*	877.72 ± 151.94* ^,#^	1570.55 ± 292.23* ^,#,†^	158.28	< 0.001
AUC	3058.38 ± 194.72	2949.88 ± 153.25	562.50 ± 73.38*	959.50 ± 114.43* ^,#^	1223.25 ± 166.70* ^,#,†^	507.50	< 0.001

PI: peak intensity; WIS: wash-in slope; AUC: area under the curve. * *P*  < 0.05 compared with the Con group; ^#^ *P*  < 0.05 compared with the As _2_O _3_ group; and ^†^ *P*  < 0.05 compared with the Dan-L group.

To further assess the cardioprotective effect of dantrolene, myocardial tissues were collected for immunofluorescence staining of platelet-endothelial cell adhesion molecules (CD31/PECAM-1) ( Figure 1). CD31 is a cell membrane surface glycoprotein that plays a significant role in angiogenesis and is expressed mainly on the surfaces of endothelial cells, macrophages, and platelets [12]. Blue fluorescence was used to label the cell nuclei, whereas green fluorescence was used to indicate the expression of CD31 in the cells. In the As _2_O _3_ group, many cell nuclei were stained blue, which was attributed to the infiltration of various cells, such as inflammatory cells, fibroblasts and endothelial cells. Moreover, disordered and unevenly sized capillaries were visible, and the CD31 absorbance value increased. After the application of Dan, the number of infiltrating cells decreased, and the absorbance value of CD31 also decreased. Moreover, the decrease was more significant in the Dan-H group ( *P*  < 0.05; Figure 2A). In combination with the findings of MCE, these findings suggest that arsenic can damage the endothelial cells of capillaries, thereby disrupting microcirculation blood perfusion in the myocardium. Different doses of Dan may alleviate, to varying degrees, the damage induced by As _2_O _3_ to myocardial microcirculatory perfusion.

**Figure 1:**
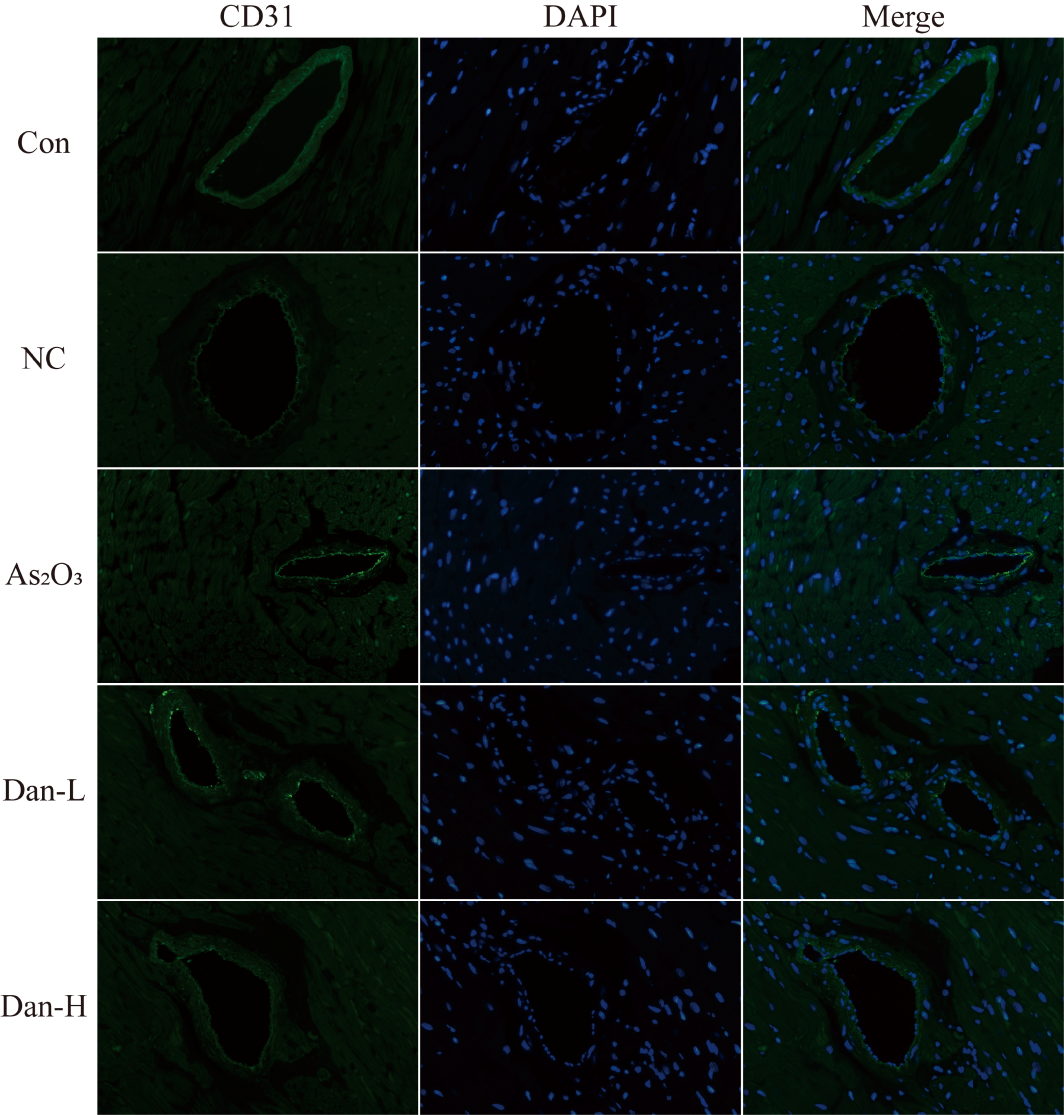
Figure 1 Representative CD31 immunofluorescence images depicting the myocardial tissue in each group of rats (×400) CD31 (green) and DNA (blue).

**Figure 2:**
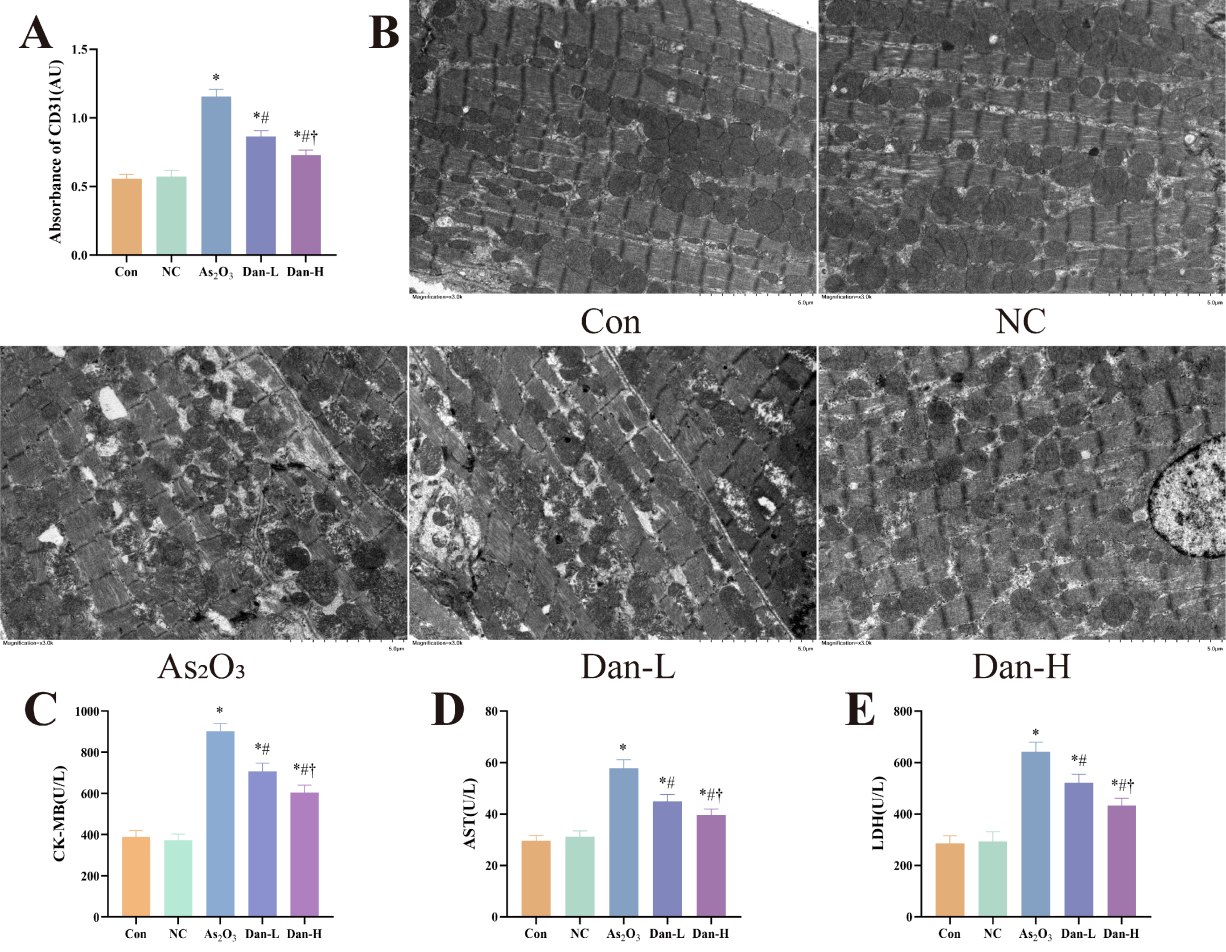
Figure 2 CD31 content in the myocardium, myocardial electron microscopy images and levels of myocardial injury markers in each group of rats (A) Optical density measurements of CD31 immunofluorescence in different groups of rats. (B) Electron microscopy images showing the myocardial microstructure of each group of rats (×3000). (C) CK-MB: creatine kinase. (D) AST: glutamate transaminase. (E) LDH: lactate dehydrogenase. *P < 0.05 compared with the Con group; #P < 0.05 compared with the As2O3 group; and †P < 0.05 compared with the Dan-L group.

To further explore the experimental findings, transmission electron microscopy was performed to evaluate the myocardial cellular morphology in all the rat groups, and the serum levels of myocardial injury biomarkers were quantitatively analyzed. Electron microscopic analysis revealed that both the Con group and the NC group exhibited normal and well-organized myocardial myofibrillar architecture, with morphologically intact and numerically abundant mitochondria. In contrast, the As _2_O _3_ group presented disrupted myocardial myofibrillar bundles, with partial dissolution and fragmentation of myofibrils, distorted Z lines, and indistinct boundaries between light and dark bands. The mitochondria exhibited a disorganized arrangement, with a marked reduction or complete loss of normal cristae, and in some cases, mitochondrial deformation and progressive swelling leading to rupture were observed. However, these pathological alterations were ameliorated to varying degrees in both the Dan-L and Dan-H groups ( Figure 2B). Similarly, compared with those in the Con group, the levels of CK-MB, AST and LDH in the As _2_O _3_, Dan-L and Dan-H groups were elevated. Among them, the changes in the As _2_O _3_ group were the most significant, and the changes in the Dan-H group were the smallest ( *P*  < 0.05; Figure 2C–E).

In conclusion, the data from this study confirm that arsenic exposure can cause damage to the myocardial tissue of rats, ultimately leading to cardiac dysfunction, whereas dantrolene has a protective effect to some extent on the cardiac dysfunction caused by arsenic exposure. Although the specific mechanism of action remains to be further clarified, these findings provide a novel therapeutic strategy for cardiac dysfunction associated with arsenic exposure.

## References

[1] Patel KS, Pandey PK, Martín-Ramos P, Corns WT, Varol S, Bhattacharya P, Zhu Y (2023). A review on arsenic in the environment: bio-accumulation, remediation, and disposal. RSC Adv.

[2] Sadee BA, Galali Y, Zebari SMS (2023). Toxicity, arsenic speciation and characteristics of hyphenated techniques used for arsenic determination in vegetables. a review. RSC Adv.

[3] Alamolhodaei NS, Shirani K, Karimi G (2015). Arsenic cardiotoxicity: an overview. Environ Toxicol Pharmacol.

[4] Li Y, Wan R, Liu J, Liu W, Ma L, Zhang H (2022). In silico mechanisms of arsenic trioxide-induced cardiotoxicity. Front Physiol.

[5] Zahradníková A, Pavelková J, Sabo M, Baday S, Zahradník I, Elofsson A (2025). Structure-based mechanism of RyR channel operation by calcium and magnesium ions. PLoS Comput Biol.

[6] Uchinoumi H, Nakamura Y, Suetomi T, Nawata T, Fujinaka M, Kobayashi S, Yamamoto T (2025). Structural instability of ryanodine receptor 2 causes endoplasmic reticulum (ER) dysfunction as well as sarcoplasmic reticulum (SR) dysfunction. J Cardiol.

[7] Yang HS, Choi JM, In J, Sung T, Kim YB, Sultana S (2023). Current clinical application of dantrolene sodium. Anesth Pain Med.

[8] Schmeckpeper J, Kim K, George SA, Blackwell DJ, Brennan JA, Efimov IR, Knollmann BC (2023). RyR2 inhibition with dantrolene is antiarrhythmic, prevents further pathological remodeling, and improves cardiac function in chronic ischemic heart disease. J Mol Cell Cardiol.

[9] Walweel K, Beard N, van Helden DF, Laver DR (2023). Dantrolene inhibition of ryanodine channels (RyR2) in artificial lipid bilayers depends on FKBP12.6. J Gen Physiol.

[10] Roldan P, Ravi S, Hodovan J, Belcik JT, Heitner SB, Masri A, Lindner JR (2022). Myocardial contrast echocardiography assessment of perfusion abnormalities in hypertrophic cardiomyopathy. Cardiovasc Ultrasound.

[11] Yang Y, Li Y, Li R, Wang Z (2024). Research progress on arsenic, arsenic-containing medicinal materials, and arsenic-containing preparations: Clinical application, pharmacological effects, and toxicity. Front Pharmacol.

[12] Smith CW, Nagy Z, Geer MJ, Pike JA, Patel P, Senis YA, Mazharian A (2024). LAIR-1 and PECAM-1 function via the same signaling pathway to inhibit GPVI-mediated platelet activation. Res Pract Thromb Haemost.

